# Assessment of knowledge, attitudes, and practices regarding cardiovascular diseases (CVDs) among older individuals of rural Bangladesh: findings from a face-to-face interview

**DOI:** 10.3389/fpubh.2024.1336531

**Published:** 2024-05-24

**Authors:** Abu Bakkar Siddique, Md. Shohag Hosen, Hasna Akter, Syed Mujakkir Hossain, Md. Al Mamun

**Affiliations:** ^1^Department of Public Health and Informatics, Jahangirnagar University, Savar, Dhaka, Bangladesh; ^2^Centre for Advanced Research Excellence in Public Health, Savar, Dhaka, Bangladesh; ^3^International Centre for Research, Innovation, Training and Development (ICRITD), Savar, Dhaka, Bangladesh; ^4^AMR Reference Laboratory (Research), Bangladesh Livestock Research Institute, Savar, Dhaka, Bangladesh; ^5^Health and Environmental Epidemiology Laboratory (HEEL), Jahangirnagar University, Savar, Dhaka, Bangladesh

**Keywords:** cardiovascular diseases, knowledge, attitudes, practices, older individuals, rural Bangladesh

## Abstract

**Introduction:**

Cardiovascular diseases (CVDs) stand as the foremost contributor to global mortality, claiming roughly 17.9 million lives each year, constituting 32.1% of total fatalities. Their impact is notably profound in economies such as Bangladesh, exacting a substantial economic burden. Consequently, grasping the landscape of knowledge, attitudes, and practices is essential for timely identification and prevention strategies.

**Methods:**

This cross-sectional study, carried out between January and May 2023 in the rural regions of Zirani, Savar Upazila, Dhaka, Bangladesh, utilized convenient sampling and conducted face-to-face interviews using a semi-structured questionnaire. It encompassed socio-demographic factors, as well as knowledge, attitudes, and practices concerning CVDs. Data analysis employed descriptive statistics, chi-square tests, and regression analyses, utilizing both the R programming language and SPSS (Version 26).

**Result:**

A total of 424 participants aged 60 years and above were included. The majority were male (60.8%), and the mean age was 71.21 ± 9.21 years, 57.3% were between 60 and 70 years old. Factors such as education, monthly family income, high blood pressure, diabetes, and non-smoking. Were significantly associated with higher knowledge, attitudes and practices scores.

**Conclusion:**

This study illuminates CVD-related KAP among rural Older Individuals in Bangladesh, revealing significant associations between factors such as education, monthly family income, high blood pressure, and non-smoking, with higher scores in knowledge, attitudes, and practices regarding cardiovascular health. These insights underscore the importance of addressing socio-economic factors and health behaviors in developing targeted interventions for the prevention and management of cardiovascular diseases in this demographic.

## 1 Introduction

Cardiovascular diseases (CVDs) constitute a group of illnesses that affect the heart and blood vessels, impacting their structure and functionality. This category encompasses a diverse array of conditions such as coronary artery disease, heart failure, stroke, peripheral artery disease, among others. These conditions can arise from a variety of factors, including the accumulation of fatty deposits in the arteries (atherosclerosis), hypertension, diabetes, smoking, obesity, and genetic predispositions (1, 2). Cardiovascular diseases (CVDs) stand as the primary cause of mortality worldwide, responsible for roughly 17.9 million deaths annually, making up 32.1% of all deaths in a given year (3). Projections suggest that by 2030, the global toll of CVDs could escalate to 23.6 million deaths annually. CVDs exert a profound influence on mortality rates and contribute significantly to the burden of disability and healthcare expenditures. These diseases encompass a spectrum of conditions affecting the heart and blood vessels, such as coronary artery diseases, stroke, heart failure, and hypertension (4).

Cardiovascular diseases (CVDs) carry substantial economic implications for countries, encompassing both direct healthcare expenses and indirect costs stemming from lost productivity (5). The burden of CVDs varies geographically, with a pronounced prevalence in low- and middle-income nations. Notably, approximately 80% of CVD-related deaths occur in these regions (6). Older individuals are particularly susceptible to severe complications and poorer outcomes following CVD events like heart attacks and strokes (7). In 2019, Asia accounted for approximately 58% of the global CVD deaths, with CVD being the foremost cause of mortality in the region, claiming 10.8 million lives, constituting about 35% of all deaths in Asia (3). Over the period from 1990 to 2019, Asia experienced a notable surge in CVD-related deaths, escalating from 5.6 million to 10.8 million (3, 5).

The populations primarily impacted are those affecting from low- and middle-income nations such as Bangladesh, where approximately 80% of these fatalities are concentrated. Projections indicate that between 2011 and 2025, these countries are anticipated to suffer cumulative economic losses totaling $7.28 trillion due to all non-communicable diseases, with CVDs contributing nearly half of this estimated loss. Given this context, CVDs are recognized as a significant global public health issue (8). In Bangladesh, non-communicable disease (NCDs) are responsible for 67% of all deaths; and an estimated 30% of the total deaths are caused by CVDs (9). In Bangladesh, the prevalence of CVDs is increasing, and it is a major contributor to the burden of disease (10). Individuals aged 60 years and above, accounts for approximately 9.28% of the total population in Bangladesh, which corresponds to over 25 million people (11). The Older Individuals population in rural areas of Bangladesh is notably susceptible due to the natural aging process and the presence of associated risk factors, including hypertension, diabetes, and a sedentary lifestyle. Additionally, their lower economic status further exacerbates these challenges (12, 13). CVDs, including ischemic heart disease and stroke, have witnessed a rising prevalence in Bangladesh over the past few decades (14).

Understanding the knowledge surrounding risk factors, symptoms, and preventive measures related to Cardiovascular Diseases (CVDs) is crucial for early detection, management, and prevention (15). Attitudes toward CVDs significantly shape individual behavior, impacting adherence to recommended lifestyle changes, medication regimens, and engagement in preventive practices. Positive attitudes toward CVD prevention, regular medical check-ups, and adherence to prescribed treatments can result in better management and improved health outcomes. Conversely, negative attitudes or misconceptions may impede the adoption of healthy behaviors (16). Investigating the attitudes of older individuals toward CVDs can provide valuable insights into their perceptions, beliefs, and potential barriers to effective prevention and management (17). To promote these positive attitudes, public health campaigns should underscore the benefits of prevention, raise awareness about the importance of regular check-ups, and offer education on the long-term advantages of treatment adherence (18).

Implementing effective prevention measures against cardiovascular diseases (CVDs), including lifestyle adjustments, physical activity, dietary habits, medication adherence, and healthcare-seeking behavior, alongside initiatives promoting heart-healthy diets and discouraging tobacco use, significantly contributes to better CVD outcomes (19). Assessing the current practices of older adults in rural Bangladesh is essential for identifying areas requiring improvement, facilitating the development of targeted interventions to promote healthy behaviors and improve CVD management (20, 21). Furthermore, understanding the knowledge, attitudes, and practices (KAP) related to CVDs among older individuals in rural Bangladesh is vital for public health (22). Bangladesh, with its predominantly rural population, is experiencing a growing burden of CVDs due to lifestyle changes and limited access to healthcare (23). Investigating the KAP of older individuals offers insights into disease understanding, prevention, and healthcare-seeking behaviors. Face-to-face interviews provide comprehensive data, informing targeted interventions to improve CVD awareness, prevention, management, and factors associated with KAP, thus benefiting rural communities and advancing public health initiatives in Bangladesh (24).

There is only one study investigating knowledge, attitude, and practice (KAP) regarding cardiovascular disease CVDs among patients with coronary artery disease in Bangladesh (25). There is no single study investigating KAP toward CVDs among rural older individuals in Bangladesh. To date, this is the first study to explore the knowledge, attitude, and practice (KAP) regarding cardiovascular disease CVDs among older Individuals people of rural Bangladesh. This study employes face-to-face interviews with a comprehensive approach to assess CVDs knowledge, prevention, and management among older individuals in rural communities. Consequently, the present study aimed to explore:

- The socio-demographic characteristics of the population.
- The level of KAP toward CVDs among older Individuals of Rural Bangladesh.
- Sex difference of each KAP item.
- Associated factors toward a high level of KAP.

These findings can provide valuable guidance for developing targeted interventions and educational initiatives aimed at enhancing public health, especially in similar settings or geographical areas facing comparable challenges in CVDs prevention and management.

## 2 Materials and methods

### 2.1 Study area

A cross-sectional study was conducted on older people in rural areas of Zirani, Savar Upazila, Dhaka, Bangladesh, from January to May 2023. Convenient (non-probability) sampling was utilized, and data was collected through face-to-face interviews at participants’ residential houses using a semi-structured questionnaire, which was designed based on previous studies.

### 2.2 Sample size

The sample size was calculated using the following equation (26, 27):

**Table:** 

$n=\frac{z^{2}pq}{d^{2}};n=\frac{1.96^{2}\times 0.5\times \left(1-0.5\right)}{0.05^{2}}=384.16\approx 384$	Here, *n* = Sample size zα/2 = 1.96 (95% confidence level) *p* = prevalence estimate (50% or 0.5) (as no study found in Bangladesh among Older Individuals) *q* = (1-*p*) *d* = Precession of the prevalence estimate (10% of 0.05)

We anticipated that the prevalence estimate (*p*) in the present study would be 50%. Calculating for a 10% non-response rate, we estimated a sample size of 423 individuals. However, our actual sample size surpassed this estimate (26, 27). However, 424 data was taken for analysis for increasing the study strength.

### 2.3 Study design, participants, and procedure

The study utilized a cross-sectional survey design conducted through face-to-face interviews from January to May 2023. Convenient (non-probability) sampling was employed to enroll participants, resulting in an initial sample of 451 individuals (28). Following the exclusion of incomplete responses/missing data, a comprehensive analysis was conducted on 424 surveys/questionnaires. The data gathering utilized a paper-based semi-structured questionnaire written in Bangla, the participants’ native language. Preceding the main survey, a pilot test was administered involving 30 participants from the target demographic to evaluate the questionnaire’s clarity and acceptability. Minor modifications were implemented to the questionnaire based on feedback from the pilot test. An informed written consent statement outlining the study’s objectives, procedures, and the participants’ right to decline participation was affixed to the initial page of the questionnaire. Prior to initiating the survey, participants were asked to obtain informed written consent (i.e., *“Are you willing to participate in this study voluntarily and spontaneously?”*). The inclusion criteria of the participants included: (i) individuals had to be aged ≥60 years (13), (ii) Bangladeshi resident, (iii) be rural dwellers (iv) native language was Bangla. The participants below 60 years were excluded at the time of the interview. As CVDs are sensitive issue, the data was collected only by expert research assistants and strict confidentiality was maintained. Our principal investigator, an experienced expert in this field, oversaw the entire research process, ensuring its quality and integrity.

### 2.4 Measures

The interview sessions utilized a questionnaire comprising informed consent and four sections: socio-demographic information and KAP-related details, to gather data.

#### 2.4.1 Socio-demographic information

Some questions related to socio-demographics were asked during the survey including age (mean age and standard deviation was given at result section), sex (male/female) (26), educational status (illiterate, primary education, secondary education, higher secondary and honors level) (29), marital status (married,widowed, divorced, and unmarried), family monthly income: less than 15000BDT (Lower SES[SES=Socioeconomic status]), 15,000 to 30000BDT (Middle SES), and more than 30000BDT (higher SES) (26), employment status (yes/no), high blood pressure (yes/no), family history of high blood pressure (yes, no) (30), diabetes (yes, no), family history of diabetes (yes, no), smoking (yes, no), alcohol intake (yes, no), physical activity (yes, no), BMI (under Weight, over weight and normal weight) (27). Height and weight were measured by scales and then BMI was calculated.

#### 2.4.2 Knowledge, attitudes and practices

Knowledge, attitudes, and practices toward the CVD were measured using a total of 31 items structured questions (including 10-item for knowledge, 10-item for attitudes and 11-item for practices) based on the prior studies (21, 31–34). We collected the KAP Items from those studies. The skewness and kurtosis of all total scores were between ±2.

The knowledge section comprised of 10-item of questions having response options “Yes,” “No” and “Do not know” questions concerning personal experience and awareness (4-item), perception and knowledge (6-item) (e.g., *“Have you known about any family member, friend of neighbors who suffered from cardiovascular disease?”*) (see the items with sex difference in Table 1). The correct answer (‘Yes’) was coded as 1, while the incorrect answer and uncertainty was coded as 0 (26). The overall score ranged from 0 to 10, with an overall greater score indicates more favorable knowledge toward the CVD. The Cronbach Alpha for the knowledge items were 0.79.

**Table 1:** Distribution of each knowledge item and its sex difference.

Variables	Overall *n* (%)	Male *n* (%)	Female *n* (%)	*p*-value^*^
Have you known about any family member, friend of neighbors who suffered from cardiovascular disease?				
No	83 (19.6%)	49 (19.0%)	34 (20.5%)	0.302
Do not know	45 (10.6%)	23 (8.9%)	22 (13.3%)	
Yes	296 (69.8%)	186 (72.1%)	110 (66.3%)	
Do you think cardiovascular disease is a major health concern in Bangladesh?				
No	45 (10.6%)	30 (11.6%)	15 (9.0%)	0.596
Do not know	154 (36.3%)	90 (34.9%)	64 (38.6%)	
Yes	225 (53.1%)	138 (53.5%)	87 (52.04%)	
Do you believe that eating a healthy diet can help to prevent cardiovascular disease?				
No	48 (11.3%)	29 (11.2%)	19 (11.4%)	0.980
Do not know	166 (39.2%)	102 (39.5%)	64 (38.6%)	
Yes	210 (49.5%)	127 (49.2%)	83 (50.0%)	
Do you think that regular physical activity can help to prevent cardiovascular disease?				
No	69 (16.3%)	48 (18.6%)	21 (12.7%)	0.186
Do not know	154 (36.3%)	95 (36.8%)	59 (35.5%)	
Yes	201 (47.4%)	115 (44.6%)	86 (51.8%)	
Do you know about cholesterol level?				
No	169 (39.9%)	110 (42.6%)	59 (35.5%)	0.334
Do not know	93 (21.9%)	55 (21.3%)	38 (22.9%)	
Yes	162 (38.2%)	93 (36.0%)	69 (41.6%)	
Do you think that stress can contribute to the development of cardiovascular disease?				
No	66 (15.6%)	45 (17.4%)	21 (12.7%)	0.414
Do not know	212 (50.0%)	126 (48.8%)	86 (51.8%)	
Yes	146 (34.4%)	87 (33.7%)	59 (35.5%)	
Do you know that healthcare providers can give best information about cardiovascular disease prevention from?				
No	165 (38.9%)	100 (38.8%)	65 (39.2%)	0.275
Do not know	129 (30.4%)	85 (32.9%)	44 (26.5%)	
Yes	130 (30.7%)	73 (28.3%)	57 (34.3%)	
Do you think that taking medication for high blood pressure or high cholesterol is important in preventing cardiovascular disease?				
No	99 (23.3%)	63 (24.4%)	36 (21.7%)	0.698
Do not know	151 (35.6%)	93 (36.0%)	58 (34.9%)	
Yes	174 (41.0%)	102 (39.5%)	72 (43.4%)	
Do you think that men and women are equally in danger of cardiovascular disease?				
No	145 (34.2%)	98 (38.0%)	47 (28.3%)	0.123
Do not know	157 (37.0%)	90 (34.9%)	67 (40.4%)	
Yes	122 (28.8%)	70 (27.1%)	52 (31.3%)		Variables	Overall *n* (%)	Male *n* (%)	Female *n* (%)	*p*-value^*^
Do you know any health promotion activities related to cardiovascular disease prevention?				
No	220 (51.9%)	134 (51.9%)	86 (51.8%)	0.975
Do not know	137 (32.3%)	84 (32.6%)	53 (31.9%)	
Yes	67 (15.8%)	40 (15.5%)	27 (16.3%)	

^*^Chi-square test, bold indicates significant at 5% level of significance.

The attitudes section consisted of 10-item questions regarding the positive attitudes toward the risk factors, severity and prevention of CVD with a three-point Likert scale including 1 (‘Disagree’), 2 (‘Neutral’), 3 (‘Agree’), yielding total scores ranging from 10 to 30 (35). Examples of such questions include: *“Regular exercise is important for maintaining cardiovascular health”* (see the items with sex difference in Table 2). An overall greater score indicates more positive attitudes toward the CVD. The Cronbach Alpha for the attitude’s items were 0.80.

**Table 2:** Distribution of each attitude item and its sex difference.

Variables	Overall *n* (%)	Male *n* (%)	Female *n* (%)	*p*-value^*^
Regular exercise is important for maintaining cardiovascular health.				
Disagree	18 (7.0%)	13 (7.8%)	31 (7.3%)	0.068
Neutral	88 (20.8%)	63 (24.4%)	25 (15.1%)	
Agree	305 (71.9%)	177 (68.6%)	128 (77.1%)	
Cardiovascular disease is mostly caused by genetic factors				
Disagree	119 (28.1%)	85 (32.9%)	34 (20.5%)	**0.020**
Neutral	123 (29.0%)	70 (27.1%)	53 (31.9%)	
Agree	182 (42.9%)	103 (39.9%)	79 (47.6%)	
It is urgent to make lifestyle changes to prevent cardiovascular disease.				
Disagree	80 (18.9%)	63 (24.4%)	17 (10.2%)	**0.001**
Neutral	147 (34.7%)	89 (34.5%)	58 (34.9%)	
Agree	197 (46.5%)	106 (41.1%)	91 (54.8%)	
I am willing to make changes to my diet to prevent cardiovascular disease.				
Disagree	71 (16.7%)	54 (20.9%)	17 (10.2%)	**0.016**
Neutral	181 (42.7%)	105 (40.7%)	76 (45.8%)	
Agree	172 (40.6%)	99 (38.4%)	73 (44.0%)	
Preventing cardiovascular disease is a priority for me.				
Disagree	183 (43.2%)	119 (46.1%)	64 (38.6%)	0.307
Neutral	137 (32.3%)	79 (30.6%)	58 (34.9%)	
Agree	104 (24.5%)	60 (23.3%)	44 (26.5%)	
I believe that cardiovascular disease is a serious health issue in my community.				
Disagree	49 (11.6%)	38 (14.7%)	11 (6.6%)	**0.024**
Neutral	107 (25.2%)	67 (26.0%)	40 (24.1%)	
Agree	268 (63.2%)	153 (59.3%)	115 (69.3%)	
I am willing to take medication prescribed by a doctor to prevent cardiovascular disease.				
Disagree	107 (25.2%)	75 (29.1%)	32 (9.3%)	**0.034**
Neutral	122 (28.8%)	65 (25.2%)	57 (34.3%)	
Agree	195 (46.0%)	118 (45.7%)	77 (46.4%)	
I am willing to make changes to my daily routine to incorporate physical activity.				
Disagree	72 (17.0%)	49 (19.0%)	23 (13.9%)	0.360
Neutral	216 (50.9%)	130 (50.4%)	86 (51.8%)	
Agree	136 (32.1%)	79 (30.6%)	57 (34.3%)	
I feel confident that I can make the necessary changes to prevent cardiovascular disease.				
Disagree	83 (19.6%)	63 (24.4%)	20 (12.0%)	**0.001**
Neutral	203 (47.9%)	108 (41.9%)	95 (57.2%)	
Agree	138 (32.5%)	87 (33.7%)	51 (30.7%)	
Preventing cardiovascular disease is a shared responsibility between individuals, healthcare providers and the community.				
Disagree	85 (20.0%)	61 (23.6%)	24 (14.5%)	**0.049**
Neutral	99 (23.3%)	61 (23.6%)	38 (22.9%)	
Agree	240 (56.6%)	136 (52.7%)	104 (62.7%)	

^*^Chi-square test, bold value indicates significant at 5% level of significance.

The practices section included 11-item questions concerning individual’s practices and behaviors related to cardiovascular disease prevention with a three-point Likert scale ranging from 0 (‘Never’) to 2 (‘Always’). Examples of such questions include: *“I engage in physical activity for at least 30 min on most days of the week”* (see the items with sex difference in Table 3). Eleven practice items’ total score ranges from 0 to 22, with an overall greater score indicates more better practices and behaviors related to CVDs (35). The Cronbach Alpha for the practice’s items were 0.82.

**Table 3:** Distribution of each practice item and its sex difference.

Variables	Overall *n* (%)	Male *n* (%)	Female *n* (%)	*p*-value^*^
I engage in physical activity for at least 30 min on most days of the week.				
Never	64 (15.1%)	38 (14.7%)	26 (15.7%)	0.336
Sometimes	245 (57.8%)	156 (60.5%)	89 (53.6%)	
Always	115 (27.1%)	64 (24.8%)	51 (30.7%)	
I eat a diet that is low in saturated and trans fats and high in fruits, vegetables, and whole grains.				
Never	132 (31.1%)	82 (31.8%)	50 (30.1%)	0.682
Sometimes	226 (53.3%)	139 (53.9%)	87 (52.4%)	
Always	66 (15.6%)	37 (14.3%)	29 (17.5%)	
I limit my intake of salt and processed foods.				
Never	177 (41.7%)	116 (45.0%)	61 (36.7%)	0.143
Sometimes	171 (40.3%)	102 (39.5%)	69 (41.6%)	
Always	76 (17.9%)	40 (15.5%)	36 (21.7%)	
I avoid or limit my intake of alcohol and tobacco products.				
Never	102 (24.1%)	73 (28.3%)	29 (17.5%)	**0.001**
Sometimes	164 (38.7%)	116 (45.0%)	48 (28.9%)	
Always	158 (37.3%)	69 (26.7%)	89 (53.6%)	
I have regular check-ups with my healthcare provider to monitor my blood pressure and cholesterol levels.				
Never	166 (39.2%)	109 (42.2%)	57 (34.3%)	0.261
Sometimes	212 (50.0%)	123 (47.7%)	89 (53.6%)	
Always	46 (10.8%)	26 (10.1%)	20 (12.0%)	
I take medication as prescribed by my healthcare provider for high blood pressure or high cholesterol.				
Never	206 (48.6%)	138 (53.5%)	68 (41.0%)	**0.033**
Sometimes	155 (36.6%)	83 (32.2%)	72 (43.4%)	
Always	63 (14.9%)	37 (14.3%)	26 (15.7%)	
I practice stress-reduction techniques such as meditation, deep breathing or yoga.				
Never	229 (54.0%)	149 (57.8%)	80 (48.2%)	0.090
Sometimes	175 (41.3%)	100 (38.8%)	75 (45.2%)	
Always	20 (4.7%)	9 (3.5%)	11 (6.6%)	
I make efforts to maintain a healthy weight.				
Never	145 (34.2%)	97 (37.6%)	48 (28.9%)	0.179
Sometimes	218 (51.4%)	125 (48.4%)	93 (56.0%)	
Always	61 (14.4%)	36 (14.0%)	25 (15.1%)	
I avoid my intake of sugary beverages and foods high in added sugars.				
Never	168 (39.6%)	116 (45.0%)	52 (31.3%)	**0.001**
Sometimes	175 (41.3%)	106 (41.1%)	69 (41.6%)	
Always	81 (19.1%)	36 (14.0%)	45 (27.1%)	
I try to incorporate physical activity into my daily routine, such as taking the stairs instead of the elevator or walking instead of driving.				
Never	123 (29.0%)	79 (30.6%)	44 (26.5%)	0.349
Sometimes	239 (56.4%)	146 (56.6%)	93 (56.0%)	
Always	62 (14.6%)	33 (12.8%)	29 (17.5%)	
I use telemedicine services for the treatment of cardiovascular disease (CVD)				
Never	129 (30.4%)	80 (31.0%)	49 (29.5%)	0.896
Sometimes	190 (44.8%)	116 (45.0%)	74 (44.6%)	
Always	105 (24.8%)	62 (24.0%)	43 (25.9%)	

^*^Chi-square test, bold value indicates significant at 5% level of significance.

### 2.5 Statistical analysis

The data underwent comprehensive statistical analysis using a variety of software tools. Initial tasks including cleaning, coding, and organizing were executed utilizing Microsoft Excel. Subsequently, the prepared data were imported into SPSS software, version 26.0, for the computation of descriptive statistics such as frequencies, percentages, means, and standard deviations so that we can get the general characteristics of the population. Bivariate analyses, including the Chi-square test and Fisher’s Exact test, were conducted within the SPSS to get gender differences of each KAP items. Moreover, both bivariate and multivariable linear regression analyses were carried out using the R programming language, incorporating the total scores of knowledge, attitudes, and practices as dependent variables. Bivariate linear regression was conducted to determine the associated factors with higher level of KAP score (35). Then, significant variables fount at bivariate analyses were included in the multivariable regression analysis to make better fit to data like other studies (26, 36). A significance level of *p* < 0.05 was applied uniformly across all statistical tests.

### 2.6 Ethics statement

The study protocol underwent thorough review and approval by the Biosafety, Biosecurity, and Ethical Clearance Committee at Jahangirnagar University, Savar., Dhaka-1342, Bangladesh [Ref No. BBEC, JU/M 2023/02 (36)]. All procedures carried out in this study strictly adhered to the guidelines for research involving human participants, including those outlined in the Helsinki Declaration. Prior to participation, informed written consent was obtained from each participant, explicitly outlining the study’s procedures, objectives, and the confidentiality measures in place for their information. The first page of the questionnaire included the informed consent form, which clearly outlined the study’s objectives, voluntary participation nature, and the authority under which the study was conducted. Participants were assured of their full rights to withdraw their response at any point during the interview process. Data collection was conducted anonymously, and numerical codes were utilized to maintain the confidentiality of participants’ information. They were explicitly assured of the study’s non-harmful nature due to its anonymous conduct.

## 3 Results

### 3.1 General characteristics of the participants

A total of 424 participants were included in this study, among them 60.8% were male followed by 39.2% female respondents. The mean age was 71.21 ± 9.21 years, 57.3% were between 60 and 70 years old. Among the respondents, majority (34.2%) people were illiterate followed by primary education (23.8%), secondary education (14.9%) and higher secondary education (14.9%). The lowest proportion of participants were from honors level (12.3%). Most of the participants (54.7%) had family monthly income less than 15,000 BDT [BDT = Bangladeshi Taka] with the range of less than 15,000tk, 15,000–30,000tk, and more than 30,000tk. Majority (76.2%) participants were married followed by widowed (10.8%) and divorced (5.4%). Only 7.5% were from never married category and 61.6% people were currently employed. Among 424 participants, 71% respondents had high blood pressure, 56.6% people have diabetes, 51.2% were smoker, 22.9% were consumed alcohol and 59.9% participants were regularly engaged with physical activity. Besides this, 231 participants had family history of high blood pressure and 207 participants had family history of diabetes. Among the participants, majority was the overweight (69.1%) although underweight was only 7.3% (Table 4).

**Table 4:** General characteristics of the participants (*N* = 424).

Variables	Categories	Frequency (n)	Percentage (%)
Age	60–70 years	243	57.3
	More than 70 years	181	42.7
Sex	Male	258	60.8
	Female	166	39.2
Educational status	Illiterate	145	34.2
	Primary education	101	23.8
	Secondary education	63	14.9
	Higher secondary	63	14.9
	Honors level	52	12.3
Marital status	Married	323	76.2
	Widowed	46	10.8
	Divorced	23	5.4
	Unmarried	32	7.5
Monthly income	<15,000 BDT (Low SES)	232	54.7
	15,000–30,000 BDT (Middle SES)	93	21.9
	>30,000 BDT (Higher SES)	99	23.3
Employment status	Yes	261	61.6
	No	163	38.4
High blood pressure	Yes	301	71.0
	No	123	29.0
Family history of High blood pressure	Yes	231	54.5
	No	191	45.0
Diabetes	Yes	240	56.6
	No	184	43.4
Family history of diabetes	Yes	207	48.8
	No	217	51.2
Smoking	Yes	217	51.2
	No	207	48.8
Alcohol intake	Yes	97	22.9
	No	327	77.1
Physical activity	Yes	254	59.9
	No	170	40.1
BMI	Under Weight	31	7.3
	Over weight	293	69.1
	Normal weight	100	23.6

BDT, Bangladeshi Taka; 1 BDT, 0.0091 US$ in 20 October, 2023; SES, socio-economic status.

### 3.2 Knowledge regarding CVD

**Figure 1:**
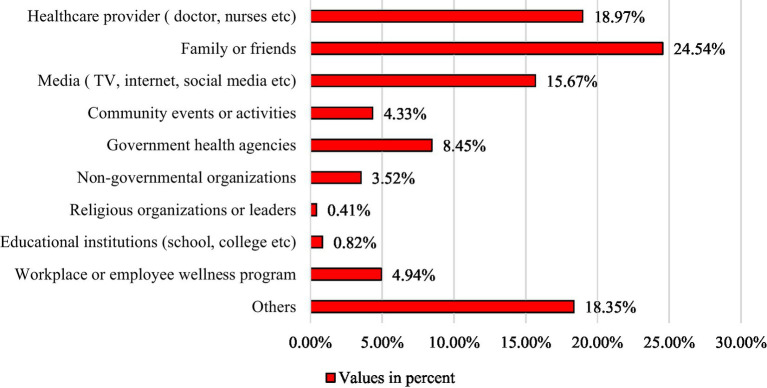
Source of information about CVDs.

The average score for the knowledge items stood at 4.08 (with a standard deviation of 2.84) out of 10, representing an overall accuracy rate of merely 40.80%. Table 1 illustrates the distribution of each knowledge item and any disparities between sexes. Multiple linear regression analysis revealed several factors that positively influenced the knowledge score, namely: (i) having education (for secondary education [*ꞵ* = 0.27, *p* < 0.001],for higher secondary level [*ꞵ* = 0.21, *p* < 0.001], and for honors level [ *ꞵ* = 0.34, *p* = <0.001] in reference to ‘illiterate’), (ii) high family income ( *ꞵ* = 0.10, *p* = 0.048) in reference to ‘lower family income’, (iii) having high blood pressure ( *ꞵ* = 0.18, *p* = <0.001) in reference to ‘no’ (iv) non-smoker ( *ꞵ* = 0.08, *p* = 0.005) in reference to ‘smoker’ (Table 5). Figure 1 illustrates the sources of knowledge about CVDs. Specifically, 24.54% of the respondents cited family and friends, while 18.97% identified healthcare providers as their primary sources of CVD-related information.

**Table 5:** Regression analysis predicting knowledge.

Variables	Overall	Bivariate regression analysis					Multivariable regression analysis				
	Mean (SD)	*B*	*SE*	*t*	*ꞵ*	*p*-value	*B*	*SE*	*t*	*ꞵ*	*p*-value
Age		−0.00	0.01	−0.13	−0.01	0.894	⸻	⸻	⸻	⸻	⸻
Sex											
Male	3.77 (2.84)	Ref.					Ref.				
Female	4.58 (2.80)	0.81	0.28	2.91	0.14	**0.004**	0.47	0.29	1.59	0.08	0.112
Marital status											
Married	3.95 (2.84)	Ref.					Ref.				
Widowed	4.74 (3.01)	0.79	0.44	1.77	0.08	0.078	⸻	⸻	⸻	⸻	⸻
Divorced/widowed	4.74 (2.77)	0.79	0.61	1.29	0.06	0.198	⸻	⸻	⸻	⸻	⸻
Unmarried	4.09 (2.73)	0.14	0.52	0.28	0.01	0.781	⸻	⸻	⸻	⸻	⸻
Education											
Illiterate	3.06 (2.66)	Ref.					Ref.				
Primary education	3.06 (2.56)	0.38	0.34	1.12	0.05	0.264	0.51	0.31	1.62	0.07	0.106
Secondary education	5.63 (2.54)	2.57	0.39	6.45	0.32	**<0.001**	2.21	0.31	5.60	0.27	**<0.001**
Higher secondary	4.52 (2.57)	1.46	0.39	3.66	0.18	**<0.001**	1.73	0.39	4.45	0.21	**<0.001**
Honors level	5.79 (2.95)	2.72	0.42	6.38	0.31	**<0.001**	3.00	0.45	6.62	0.34	**<0.001**
Monthly family income											
<15,000 BDT	3.50 (2.83)	Ref.					Ref.				
15,000–30,000 BDT	4.04 (2.49)	0.54	0.33	1.63	0.07	0.104	0.05	0.30	0.16	<0.01	0.870
>30,000 BDT	5.52 (2.74)	2.01	0.32	6.15	0.30	**<0.001**	0.71	0.36	1.99	0.10	**0.048**
Employment status											
Employed	3.91 (2.84)	Ref.					Ref.				
Unemployed	4.37 (2.85)	0.45	0.28	1.61	0.07	0.109	⸻	⸻	⸻	⸻	⸻
High blood pressure											
Yes	4.47 (2.94)	1.31	0.29	4.40	0.20	**<0.001**	1.13	0.31	3.65	0.18	**<0.001**
No	3.15 (2.37)	Ref.					Ref.				
Family history of high blood pressure											
Yes	4.61 (2.91)	1.17	0.27	4.29	0.20	**<0.001**	0.42	0.27	1.51	0.07	0.132
No	3.15 (2.37)	Ref.					Ref.				
Diabetes											
Yes	4.45 (2.93)	0.82	0.27	2.99	0.14	**0.003**	0.45	0.27	1.64	0.07	0.102
No	3.62 (2.68)	Ref.					Ref.				
Family history of diabetes											
Yes	4.51 (2.86)	0.82	0.27	2.99	0.14	**0.003**	0.50	0.26	1.93	0.08	0.054
No	2.86 (2.79)	Ref.					Ref.				
Smoking											
Yes	3.48 (3.48)	Ref.					Ref.				
No	4.72 (2.69)	1.23	0.27	4.57	0.21	**<0.001**	1.41	0.27	5.09	0.24	**<0.001**
Alcohol intake											
Yes	3.68 (2.70)	Ref.									
No	4.21 (2.89)	0.52	0.32	1.60	0.07	0.109	⸻	⸻	⸻	⸻	⸻
Regular walking or exercise											
Yes	4.31 (3.03)	0.55	0.28	1.98	0.09	0.048	⸻	⸻	⸻	⸻	⸻
No	3.75 (2.56)	Ref.									
BMI											
Underweight	3.77 (2.93)	Ref.									
Normal	4.27 (3.01)	0.21	0.49	0.85	0.07	0.399	⸻	⸻	⸻	⸻	⸻
Overweight	4.06 (2.79)	−0.14	0.28	0.53	0.04	0.599	⸻	⸻	⸻	⸻	⸻

B, unstandardized regression coefficient; SE, standard error; β, standardized regression coefficient; Bold indicates significant; ^†^*F*_(8,413)_ = 22.18; *p* < 0.001, *R*^2^_Adj_ = 0.287.

### 3.3 Attitudes regarding CVDs

The average score for attitude items stood at 22.49 (standard deviation = 4.35) out of 30, reflecting an overall accuracy of 74.96%. Table 2 illustrates the distribution of each attitude item and its sex discrepancy. According to multiple linear regression analysis, factors that positively forecasted attitude scores comprised: (i) having education (for secondary education [*ꞵ* = 0.21, *p* < 0.001], for higher secondary level [*ꞵ* = 0.20, *p* < 0.001], and for honors level [*ꞵ* = 0.24, *p* = < 0.001] in reference to ‘illiterate’), (ii) high family income (*ꞵ* = 0.16, *p* = 0.002) in reference to ‘lower family income’, (iii) having family history of high blood pressure (*ꞵ* = 0.13, *p* = 0.005) in reference to ‘no’, (iv) having diabetes (*ꞵ* = 0.11, *p* = 0.018) in reference to ‘no’, (iv) non-smoker (*ꞵ* = 0.22, *p* = <0.001) in reference to ‘smoker’ (Table 6).

**Table 6:** Regression analysis predicting attitudes.

Variables	Overall	Bivariate regression analysis					Multivariable regression analysis				
	Mean (SD)	*B*	*SE*	*t*	*ꞵ*	*p*-value	*B*	*SE*	*t*	*ꞵ*	*p*-value
Age		−0.03	0.02	−1.41	−0.06	0.159	⸻	⸻	⸻	⸻	⸻
Sex											
Male	21.91 (4.68)	Ref.									
Female	23.40 (3.62)	1.48	0.42	3.47	0.16	**0.001**	1.09	0.46	2.35	0.12	0.019
Marital status											
Married	22.50 (4.54)	Ref.									
Widowed	22.85 (3.49)	0.34	0.68	0.50	0.02	0.618	⸻	⸻	⸻	⸻	⸻
Divorced/widowed	22.35 (4.01)	−0.15	0.94	−0.17	−0.00	0.868	⸻	⸻	⸻	⸻	⸻
Unmarried	21.97 (3.89)	−0.53	0.80	−0.66	−0.03	0.508	⸻	⸻	⸻	⸻	⸻
Education											
Illiterate	21.03 (4.59)										
Primary education	21.56 (4.51)	0.53	0.53	1.01	0.05	0.314	0.61	0.50	1.21	0.05	0.226
Secondary education	24.60 (3.61)	3.57	0.61	5.77	0.29	**<0.001**	2.59	0.62	4.15	0.21	**<0.001**
Higher secondary	23.62 (3.23)	2.59	0.61	4.18	0.21	**<0.001**	2.55	0.61	4.17	0.20	**<0.001**
Honors level	24.46 (3.27)	3.43	0.66	5.17	0.25	**<0.001**	3.20	0.70	4.52	0.24	**<0.001**
Monthly family income											
<15,000 BDT	21.64 (4.23)										
15,000–30,000 BDT	22.39 (4.52)	0.74	0.51	1.45	0.07	0.149	0.36	0.48	0.75	0.03	0.456
>30,000 BDT	24.59 (3.79)	2.94	0.50	5.84	0.28	**<0.001**	1.73	0.56	3.08	0.16	**0.002**
Employment status											
Employed	22.28 (4.61)										
Unemployed	22.83 (3.91)	0.55	0.43	1.28	0.06	0.203	⸻	⸻	⸻	⸻	⸻
High blood pressure											
Yes	22.69 (4.64)	0.67	0.46	1.44	0.07	0.150	⸻	⸻	⸻	⸻	⸻
No	22.02 (3.54)	Ref.									
Family history of high blood pressure											
Yes	23.23 (4.41)	1.64	0.41	3.93	0.18	**<0.001**	1.15	0.41	2.80	0.13	**0.005**
No	21.61 (4.11)	Ref.									
Diabetes											
Yes	23.02 (4.61)	1.20	0.42	2.85	0.13	**0.005**	1.00	0.42	2.37	0.11	**0.018**
No	21.81 (3.90)	Ref.									
Family history of Diabetes											
Yes	23.02 (4.25)	1.03	0.42	2.47	0.11	**0.014**	0.62	0.41	1.50	0.07	0.134
No	21.99 (4.40)	Ref.									
Smoking											
Yes	21.51 (4.71)	Ref.									
No	23.53 (3.69)	2.01	0.41	4.90	0.23	**<0.001**	1.99	0.44	4.45	0.22	**<0.001**
Alcohol intake											
Yes	21.72 (4.76)	Ref.									
No	22.72 (4.21)	1.00	0.50	1.99	0.09	**0.047**	1.00	0.47	2.12	0.09	**0.035**
Regular walking and exercise											
Yes	22.67 (4.48)	0.43	0.43	1.02	0.04	0.309	⸻	⸻	⸻	⸻	⸻
No	22.23 (4.17)	Ref.									
BMI											
Underweight	21.58 (4.30)	Ref.									
Normal	22.83 (4.21)	1.24	0.89	1.40	0.12	0.164	⸻	⸻	⸻	⸻	⸻
Overweight	22.47 (4.41)	0.89	0.82	1.09	0.09	0.278	⸻	⸻	⸻	⸻	⸻

B, unstandardized regression coefficient; SE, standard error; β, standardized regression coefficient; Bold indicates significant; ^†^*F*_(8,413)_ = 18.23; *p* < 0.001, *R*^2^_Adj_ = 0.246.

### 3.4 Practices regarding CVDs

The average score for the practice items stood at 9.14 (with a standard deviation of 4.53) out of 22, implying an overall correctness rate of merely 41.54%. Table 3 illustrates the distribution of each practice item and its disparity between sex. According to the multiple linear regression analysis, factors that positively predicted attitude scores were: (i) having education (for secondary education [*ꞵ* = 0.23, *p* < 0.001], for higher secondary level [*ꞵ* = 0.23, *p* < 0.001], and for honors level [*ꞵ* = 0.32, *p* = < 0.001] in reference to ‘illiterate’), (ii) high family income (*ꞵ* = 0.17, *p* = 0.001) in reference to ‘lower family income’, (iii) having diabetes (*ꞵ* = 0.13, *p* = 0.003) in reference to ‘no’, (iv) non-smoker (*ꞵ* = 0.22, *p* = <0.001) in reference to ‘smoker’, (v) alcohol intake (*ꞵ* = 0.09, *p* = 0.035) in reference to ‘non-alcoholic,’ (vi) regular walking/exercise (*ꞵ* = 0.13, *p* = 0.026) in reference to ‘no’ (Table 7).

**Table 7:** Regression analysis predicting practices.

Variables	Overall	Bivariate regression analysis					Multivariable regression analysis				
	Mean (SD)	*B*	*SE*	*t*	*ꞵ*	*p*-value	*B*	*SE*	*t*	*ꞵ*	*p*-value
Age		−0.04	0.02	−1.81	−0.08	0.070	⸻	⸻	⸻	⸻	⸻
Sex											
Male	8.57 (4.61)	Ref.					Ref.				
Female	10.04 (4.27)	1.47	0.44	3.30	0.15	**0.001**	0.42	0.48	0.87	0.04	0.385
Marital status											
Married	8.86 (4.59)	Ref.					Ref.				
Widowed	10.98 (1.61)	2.12	0.70	2.99	0.14	**0.003**	2.30	0.64	3.57	0.15	**<0.001**
Divorced/widowed	9.74 (3.78)	0.88	0.97	0.91	0.04	0.364	1.20	0.88	1.36	0.05	0.175
Unmarried	8.94 (4.02)	0.07	0.83	0.10	0.00	0.924	−0.01	0.76	−0.02	−0.01	0.981
Education											
Illiterate	7.34 (4.11)	Ref.					Ref.				
Primary education	8.18 (4.26)	0.84	0.54	1.55	0.07	0.121	0.78	0.51	1.53	0.07	0.126
Secondary education	11.16 (3.41)	3.82	0.63	6.06	0.30	**<0.001**	2.94	0.64	4.58	0.23	**<0.001**
Higher secondary	10.32(4.21)	2.97	0.63	4.73	0.23	**<0.001**	2.81	0.65	4.33	0.22	**<0.001**
Honors level	12.17 (4.93)	4.83	0.67	7.16	0.35	**<0.001**	4.42	0.72	6.10	0.32	**<0.001**
Monthly family income											
<15,000 BDT	8.24 (4.44)	Ref.					Ref.				
15,000–30,000 BDT	8.89 (4.19)	0.65	0.53	1.22	0.05	0.223	0.57	0.50	1.15	0.05	0.251
>30,000 BDT	11.48 (4.26)	3.24	0.52	6.22	0.30	**<0.001**	1.90	0.57	3.31	0.17	**0.001**
Employment status											
Employed	8.89 (4.69)	Ref.					Ref.				
Unemployed	9.54 (4.25)	0.64	0.45	1.43	0.06	0.153	⸻	⸻	⸻	⸻	⸻
High blood pressure											
Yes	9.25 (1.68)	0.38	0.48	0.79	0.03	0.431	⸻	⸻	⸻	⸻	⸻
No	8.87 (4.04)	Ref.					Ref.				
Family history of high blood pressure											
Yes	9.56 (4.51)	0.92	0.44	2.10	0.10	**0.036**	0.51	0.41	1.27	0.05	0.207
No	8.63 (4.54)	Ref.					Ref.				
Diabetes											
Yes	9.59 (4.69)	1.02	0.44	2.33	0.11	**0.021**	1.25	0.41	3.02	0.13	**0.003**
No	8.56 (4.27)	Ref.					Ref.				
Family history of Diabetes											
Yes	9.52 (4.41)	0.73	0.43	1.67	0.08	0.096	⸻	⸻	⸻	⸻	⸻
No	8.78 (4.63)	Ref.					Ref.				
Smoking											
Yes	8.11 (4.66)	Ref.					Ref.				
No	10.23 (4.14)	2.12	0.42	4.95	0.23	**<0.001**	2.08	0.45	4.56	0.23	**<0.001**
Alcohol intake											
Yes	7.92 (4.20)	Ref.					Ref.				
No	9.50 (4.57)	1.58	0.51	3.06	0.07	**0.002**	1.00	0.47	2.12	0.06	0.051
Regular walking/exercise											
Yes	9.69 (4.66)	1.35	0.44	3.05	0.14	**0.002**	0.88	0.39	2.24	0.09	**0.026**
No	8.33 (4.23)	Ref.									
BMI											
Underweight	8.32 (4.35)	Ref.					Ref.				
Normal	9.69 (5.11)	1.36	0.93	1.47	0.12	0.143	⸻	⸻	⸻	⸻	⸻
Overweight	9.04 (4.34)	0.71	0.85	0.84	0.07	0.402	⸻	⸻	⸻	⸻	⸻

B, unstandardized regression coefficient; SE, standard error; β, standardized regression coefficient; Bold indicates significant; ^†^*F*_(9,413)_ = 20.64; *p* < 0.001, *R*^2^_Adj_ = 0.271.

## 4 Discussion

Cardiovascular diseases (CVDs) stand as the primary contributor to worldwide mortality, notably impacting low- and middle-income countries (37, 38). South Asia bears the brunt of this burden due to its substantial population and the early onset of diseases (39). Implementing precise population-based approaches holds the potential to mitigate the morbidity and mortality linked with CVDs by tackling modifiable risk factors (40, 41). Enhanced understanding of CVDs and their risk elements is instrumental in fostering healthier lifestyles (21, 42). This research delves into the knowledge, attitudes, and practices regarding CVDs among older individuals in rural Bangladesh, pinpointing associated factors.

Our recent research identified several variables, including education, monthly family income, high blood pressure, and smoking, that showed significant associations with knowledge of cardiovascular diseases (CVDs). Notably, individuals with honors-level education exhibited superior knowledge of CVDs compared to those who were illiterate. This finding aligns with previous studies that have also demonstrated a correlation between higher education levels and better understanding of CVDs (21, 31, 43). It stands to reason that individuals with greater educational attainment are exposed to more information about diseases, thereby fostering higher CVDs knowledge scores (18).

The study found that respondents with a monthly family income greater than 30,000 BDT had higher knowledge of CVDs compared to those with a monthly income less than 15,000 BDT. This result aligns with some previous studies which also participants with higher income possess better CVDs-related knowledge (17, 20, 21, 43). This may be due to the fact that higher income leads to a better lifestyle and consequently higher CVDs’ knowledge.

The study indicated that individuals with high blood pressure (hypertension) possessed a more comprehensive understanding of cardiovascular diseases (CVDs) compared to those without hypertension. This phenomenon may stem from the inclination of individuals with CVDs to educate themselves about the condition. Interestingly, this finding contradicted the results of another study, which revealed that individuals with CVDs or risk factors for CVDs exhibited lesser knowledge about the condition compared to those without such risks (32). Furthermore, our study identified a higher level of CVD-related knowledge among non-smokers in contrast to smokers. Research on smoking and its relationship to CVDs indicates that smoking significantly contributes to CVDs, accounting for approximately one in four CVD-related deaths (2). In a similar vein, a previous study examining perceptions of smoking found that 91% of male patients hospitalized due to CVDs were daily smokers and had limited awareness of the adverse effects of smoking (44). Remarkably, no prior study has explored and compared the knowledge regarding CVDs between smokers and non-smokers. Therefore, our current study promises to shed new light on this variable.

In our study, education, monthly family income, family history of blood pressure, diabetes and smoking was found to be associated with good attitudes regarding CVDs. The study revealed that participants with higher (honors) level education and monthly family income greater than 30,000 BDT had good attitude regarding CVDs compared to those with no education and monthly family income less than 15,000 BDT. This result is in line with some previous study that stated good attitude regarding CVDs was significantly associated with higher (university) education and higher socioeconomic status (31, 33). The study also found participants with family history high blood pressure had better attitude about CVDs compared to those with no family history of high blood pressure. This is in line with another study which found family history of CVDs was significantly associated with a better level of attitude CVDs (31). Our study also found participants with diabetes had a better attitude toward CVDs compared to those not affected with diabetes. The study also revealed respondents with no smoking habit had a positive attitude regarding CVDs compared to those who smoke. This result is somewhat similar to a study conducted in Kerala, India which found that over 70% of respondents had a favorable attitude toward encouraging others to give up smoking (34).

The study found marital status, education, diabetes, monthly family income, smoking, alcohol intake and regular walking to be significantly associated with good practices regarding CVDs. Widowed participants had better CVDs-related practices compared to married participants. This result differs from a study conducted in Korea which found living with a spouse is associated with healthier behavior among Korean middle-aged individuals in comparison to living alone (45). This variation could be attributed to differences in study settings, geographical areas, and study populations that may be implied different public awareness or education programs. The study found that respondents with honors-level education had better CVDs practice compared to those who had no formal education. This finding is congruent with another study which also reported participants who attended university had better dietary and smoking behavior (15, 21, 31). Given that more education can lead to more exposure to information regarding diseases, it appears logical to predict that people with higher KAP toward CVDs will also have higher education levels (31). The study maintained that participants with monthly family income greater than 30,000 BDT had better CVDs-related practices compared to those with monthly family income less than 15,000 BDT. The study also found participants suffering from diabetes had better practices toward CVDs than those without diabetes. This is consistent with some previous studies which also reported that diabetic patients have better practices toward CVDs and lifestyle behaviors compared to other groups (19, 46, 47). Moreover, they have a higher frequency of exercise per week and a lower smoking intake (48). This is likely because they have learned from physicians that these practices can improve their diabetes status and prevent cardiovascular diseases (CVDs). Engaging in regular exercise, reducing smoking, and maintaining a healthy lifestyle and diet can effectively lower the risk of CVDs (19, 46, 47). Our study found non-smoker participants had better CVDs practices compared to smokers, which is in line with another study that also reported non-smokers had better cardiovascular disease prevention practices in terms of lifestyle behaviors compared to smokers and individuals with other risk factors (16). The present study asserted that non-alcoholic participants had better practices toward CVDs compared to alcoholic participants. This result is incongruent with a previous study that stated non-drinkers were more likely to have characteristics associated with increased cardiovascular disease mortality (49). Our study also found participants who practices regular walking had better CVDs practices than participants who did not walk regularly.

In terms of sources of information, our study found 24.54% participants received CVDs related information from their family and friends, while 18.97 and 15.67% received information from healthcare providers (doctor, nurses etc.) and media (TV, internet, social media etc.) respectively. A previous study on CVDs conducted in Italy reported television and physicians were the major sources of information about CVDs among general population (30), which is incongruent to our current study. Another study reported traditional media (71%), internet-based media (45%) and community health services (23%) were major sources of information regarding CVDs, while only 20% reported health care providers as their source of information about CVDs (50). The probable cause maybe elder people have less access to an increasing variety of mobile devices and tools and they trust their friends and close-persons more than social media (51). It’s regrettable that there are currently no ongoing public health campaigns in this location, and this information will aid in the planning and implementation of new campaign programs for the area.

In essence, this study uncovers the elements shaping cardiovascular disease (CVD) understanding, perspectives, and behaviors within the older individuals of Bangladesh. It underscores the significance of education, income, and controllable risk factors such as hypertension and smoking. Moreover, it advocates for personalized health communication tactics to cater to the information preferences and trust dynamics of older rural residents. These findings offer valuable guidance for crafting focused interventions and educational initiatives aimed at enhancing public health, especially in regions burdened by high rates of disease.

### 4.1 Strengths and limitations of this study

This study’s strength lies in its novel and comprehensive exploration of cardiovascular disease (CVD) knowledge, attitudes, and practices (KAP) among older individuals in rural Bangladesh. It fills a critical gap in the literature, being the first of its kind in this demographic and geographic context. Its methodologies and findings offer a valuable template for replication in similar settings, enhancing CVD prevention and management efforts. There are several limitations to consider in this study. Firstly, the use of convenient sampling may introduce sampling bias, as the findings may not be representative of the entire Older Individuals population in rural Bangladesh. Secondly, relying on self-reporting through face-to-face interviews can lead to recall and social desirability biases, potentially affecting the accuracy of the responses. Thirdly, the cross-sectional design of the study hinders establishing causality or understanding temporal relationships between variables. Additionally, the study was conducted in a specific rural area of Bangladesh, limiting the generalizability of the findings to other rural regions or urban areas. Finally, certain variables, such as smoking, alcohol intake, and employment status, were dichotomous, thereby restricting our capacity to provide more in-depth details regarding these variables.

## 5 Conclusion and recommendations

In conclusion, this study represents the first investigation into the knowledge, attitudes, and practices (KAP) regarding cardiovascular diseases (CVDs) among older individuals in rural Bangladesh. Through this study, we aimed to explore socio-demographic characteristics, levels of KAP toward CVDs, sex differences in each KAP item, and associated factors toward a high level of KAP employing face-to-face interviews and a comprehensive questionnaire. Our findings underscore the importance of education, income, and modifiable risk factors such as high blood pressure and smoking in influencing CVD-related KAP among rural older individuals. Notably, we identified significant associations between education, income, family history of CVDs, and lifestyle behaviors with improved KAP scores. Moreover, our study sheds light on the sources of information about CVDs, with family and friends emerging as prominent influencers. Ultimately, these insights can guide the development of targeted interventions and education programs aimed at enhancing public health outcomes, particularly in regions burdened by high rates of CVDs. By addressing information preferences and trust levels among rural older individuals, tailored health communication strategies can effectively promote healthier behaviors and contribute to the prevention and management of CVDs. Recommendations derived from our study include developing specific health education programs to increase awareness about CVDs among older individuals in rural Bangladesh, encouraging ongoing education as higher education levels are linked to better CVD knowledge and attitudes, implementing measures for early detection and prevention of CVDs among older individuals, creating anti-smoking programs tailored to the older population to reduce smoking prevalence, and promoting regular physical activity through community-based initiatives and accessible facilities. These recommendations aim to address key factors influencing CVD-related KAP and contribute to improved public health outcomes in rural areas. Additionally, a more extensive longitudinal study can be recommended to address the research gaps comprehensively.

## Data availability statement

The raw data supporting the conclusions of this article will be made available by the corresponding author, without undue reservation.

## Ethics statement

The study protocol underwent thorough review and approval by the Biosafety, Biosecurity, and Ethical Clearance Committee at Jahangirnagar University, Savar., Dhaka-1342, Bangladesh [Ref No. BBEC, JU/M 2023/02 (36)]. All procedures carried out in this study strictly adhered to the guidelines for research involving human participants, including those outlined in the Helsinki Declaration. Prior to participation, informed written consent was obtained from each participant, explicitly outlining the study’s procedures, objectives, and the confidentiality measures in place for their information. The first page of the questionnaire included the informed consent form, which clearly outlined the study’s objectives, voluntary participation nature, and the authority under which the study was conducted. Participants were assured of their full rights to withdraw their response at any point during the interview process. Data collection was conducted anonymously, and numerical codes were utilized to maintain the confidentiality of participants’ information. They were explicitly assured of the study’s non-harmful nature due to its anonymous conduct. The studies were conducted in accordance with the local legislation and institutional requirements. The participants provided their written informed consent to participate in this study.

## Author contributions

AS: Conceptualization, Data curation, Formal analysis, Investigation, Methodology, Writing – original draft, Writing – review & editing. MH: Data curation, Formal analysis, Writing – original draft. HA: Investigation, Writing – original draft. SH: Investigation, Writing – original draft. MA: Conceptualization, Writing – review & editing.
